# Effects of electromagnetic fields of low frequency and low intensity on rat metabolism

**DOI:** 10.1186/1477-044X-6-3

**Published:** 2008-04-01

**Authors:** Gabriele Gerardi, Antonella De Ninno, Marco Prosdocimi, Vanni Ferrari, Filippo Barbaro, Sandro Mazzariol, Daniele Bernardini, Getullio Talpo

**Affiliations:** 1 Department of Veterinary Clinical Sciences, University of Padua, Agripolis, Viale dell'Università 16, 35020 Legnaro, Padua, Italy; 2 ENEA, CR Frascati, Dept. FIM, Via E. Fermi, 27 00044, Frascati, Rome, Italy; 3 PROMETEO S.r.l., Via Marostica 2, 35100, Padua, Italy; 4 Department of Public Health, Comparative Pathology, and Veterinary Hygiene, University of Padua, Italy

## Abstract

A series of experiments on rats have been performed, to study the effects of long time (50 days) exposure to electromagnetic fields of extremely low frequency (ELF, i.e. less than 100 Hz) and amplitude (non thermal), testing whether the metabolic processes would be affected. The background lies on recent observations on the behaviour of isolated enzymes in vitro exposed to EFL fields. In these experiments, the cyclotron (or Larmor) frequency of the metallic ion has been used to "stimulate" the metalloproteins redox-active site, thus obtaining a clear variation of the enzyme functionality. In this paper we have extended for the first time the check to more complex animal metabolism. The novelty of this approach implies that a large amount of data had to be analyzed since it was not possible, in principle, to select only a few parameters among all the potential effects. Several biochemical parameters have been evaluated by comparing their values during the periods of exposure (field ON) and non exposure (field OFF). The evidence that long term exposure to electromagnetic fields with a well defined frequency may have relevant effects on parameters such as body weight, blood glucose and fatty acid metabolism has been obtained.

## Background

Exposure to artificially generated electromagnetic (e.m.) fields is a common occurrence for a large number of individuals: its biological consequences, still largely unknown, are being studied in experimental animals [1-4] and in humans [5]. At present, the body functioning is generally formulated in terms of biomolecules and their interactions; the living state is described in terms of molecular biology. In this picture, electromagnetism barely fits. It has been believed for a long time that biological dynamics should be accounted for by classical physics, but this conceptual frame can explain neither the space-time order existing in living matter nor the selectivity of biocomponent interactions. Some of the Authors of the present paper have previously studied the living matter from a physical point of view as long-ordered coherent systems, where the electromagnetic fields play the paramount role of information transducers [6-8].

In the '80s, it has been recognized [9,10] that very weak magnetic fields applied to living organisms produce variations of the ions concentration within cells whenever the frequency of the applied field matches a characteristic frequency of the involved ion species, called ion cyclotron frequency (ICR) f_c _$f_{c}=\frac{1}{2\pi }\frac{q}{m}B_{0}$ Where *q *and *m *are respectively the electrical charge and the mass of the ion, and B_0 _is a constant magnetic field. Furthermore, according to Zhadin and co-workers [11,12] an ion current exhibits a transient enhancement by applying, parallel to the static field B, an alternating magnetic field B_ac_, which has a frequency matching the cyclotron frequency *f*_c_. Such a mechanism should be a good candidate to explaining the selective enhancement of the ions flow through the cell membranes, and would consequently affect the rate of biochemical reactions and the enzymatic activities.

More recently, these observations have been checked by applying e.m. fields to isolated enzymes studied in vitro. The cyclotron (or Larmor) frequency of the metallic ion has been used to "stimulate" the metalloproteins redox-active site [13]. The simplest coupling model toy of the motion of a single particle of mass *m *and charge *e *in a constant magnetic field of strength B_0 _leads to the superposition of a uniform precession of angular frequency *f*_*L *_(known as Larmor precession) about the direction of the field on the original motion. The Larmor precession provides a mechanism by which biological systems become sensitive to small static and resonating magnetic fields extensively described by Edmonds [14]. As clearly stated by Edmonds, the peculiarity of Larmor precession makes the existence of a resonating effect also at room temperature possible, provided that ions are considered as tightly bounded in a central force field, as they actually are, and not so free as particles in a gas-like model. Further, the perturbation introduced by the e.m. field must be negligible. This sets an upper limit to the intensity of the energy transferred to the ions by the external field and hence to the amplitude of the e.m. perturbation.

In order to investigate the possible consequences of such findings on living organisms, a series of experiments with rats exposed to fields of very low intensity and frequency were carried out, aimed at testing whether such non-thermal e.m. fields may influence the basic metabolic processes. The novelty of our approach leads to the analysis of a large amount of data since it was not possible, in principle, to select only a few parameters among all the potential targets. In these experiments, it has been observed that biochemical and morphological parameters have been affected by 50 days exposure, even though the amplitude and frequency of the field was largely under the threshold usually considered to be effective by the medical community. In order to maximise the likelihood that preliminary experiments might reveal biological effects of some relevance, aging animals were also studied, considering that adult animals may be more resistant to external interventions than aging animals.

## Methods

### Description of the apparatus

Experiments on the effects of the ELF (Extremely Low Frequency) weak electromagnetic fields have been performed by using the electronic equipment QUEC-PHISIS QPS1™, provided by PROMETEO S.r.l. (an Italian firm which has developed a device able to generate ELF field on a coil aimed at stimulating the motion of selected ionic species through the ionoresonance effect).

The electronic device being used is able to generate waves in the range where the cyclotron frequencies of the ions involved in metabolic processes lie. Moreover, it is well known from clinical impedencymetry [15] that e.m. fields belonging to a specific frequency range, peaked on 50 KHz, are able to cross the cell membrane with the maximum efficiency. Therefore, even if the information directed to the ionic flux control travels on low (less than 100 Hz) frequencies signals, higher frequency signals have to be carried on in order to affect the intra cellular environment and the ionic exchanges. To this aim, the device generates programmable patterns of signals, carrying the basic information with a superimposed amplitude modulation at 50 KHz. The signal feeds a coil or a stack of coils which, in turn, generates the magnetic field.

The magnetic field produced inside the cage was measured during the exposure with a gauss meter F.W Bell (Sypris Q3 Test and Measurements) Mod. 7010 equipped with a low field Hall sensor MOX 71-2506-05, with the resolution of 0.1 nT.

In order to study the influence of a prolonged exposure to these fields, initially one coil of 75 cm of diameter was used, which allowed the exposure of two rats in one cage positioned just in the middle of the coil Such a dimension let us to dwell easily a cage for long time, thus avoiding disturbing the animal's daily life. The field produced had a frequency of f = 31.6 Hz and an amplitude of about 40 *μ*T, in order to stimulate the Ca^2+ ^dynamics. The amplitude was set equal to the amplitude of B_0 _in order to accomplish to the range of validity of the Edmonds model.

**Figure 1:**
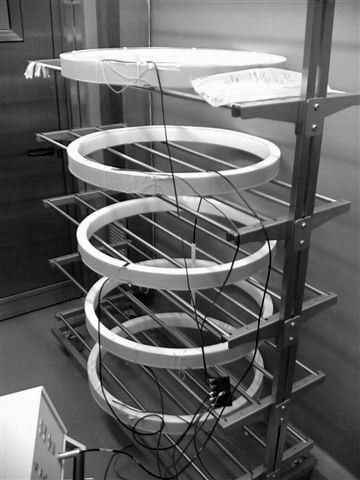
**Set of exposure.** Each coil has a diameter of 75 cm and the distance between cages is 37 cm.

In a subsequent experiment, a set of identical Helmoltz coils arranged on a rack (Fig. 1) was assembled thus allowing the simultaneous exposure of up to 16 animals housed in 5 cages. Each cage has been put on a shelf of the rack, however, it must be remembered that the Helmoltz coils set up allows to have a uniform magnetic field inside the coils provided that they are spaced of a length equal to their radius. The limitations of the maximum output power of the device (16 Watt) sets a limit to the maximum diameter of the coil. The exposure frequency was again chosen f = 31.6 Hz to stimulate the Ca^2+ ^ion dynamics. In fact the q/m ratio of Ca^2+ ^ion is 4.81 × 10^-6 ^*Coul/kg*, the corresponding f_c_/B_0 _is 0.76 (Hz/*μ*T) and the resonant frequency has been calculated being the measured geomagnetic field of 41.5 *μ*T.

**Figure 2:**
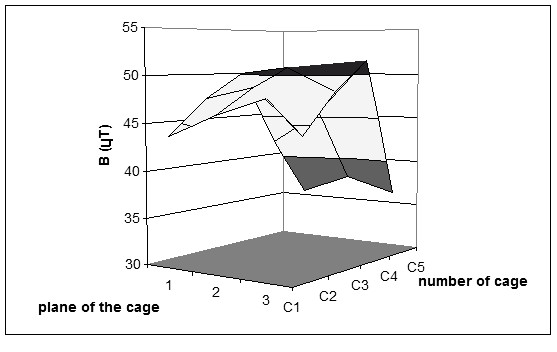
Measured static magnetic field (geo magnetic field) B_0 _along the plane of each of the 5 cages.

However, the peculiar geometry and the materials of the rack and of the grids of the cages (stainless steel) did not allow a uniform static magnetic field level to be reached neither along the ax of the rack nor on the plane of the five cages. Thus, the B_0 _magnetic field amplitude, accurately measured during the exposure, ranged between 35.5 and 51.6 *μ*T, as it can be seen in details in Fig. 2. In such a circumstance the cyclotron frequency varies along the plane of each cage accordingly to the variation of the static magnetic field. Also other ions as Co^3+ ^and Mn^3+ ^having an f_c_/B_0 _ratio quite similar to the Ca^2+ ^might have been activated.

### Animal exposure and sample collection

This Research Project was communicated on July the 7th 2005. to the Ministry of Public Health as stated by Laws (D.lgs 116/92).

The animals were kept in stabulary controlled conditions all over the duration of the experiments : temperature 21°C ± 1; hygrometry between 40% and 70%; air change per hour 8 to 12 volumes; nyctohemeral cycle 12 h/12 h; lightning 300 to 600 lux at 1 meter over the floor; noise < 60 db. The cages bases were made out of polypropylene while the ceilings were in stainless steel. In each cage up to 4 adult animals, or 3 aging animals, could be housed. A complete maintenance diet for rats (Standard Diet GLP – 4 RF 21; Charles River-Italy), administered dry *ad libitum *with water at disposal, was utilised.

In a preliminary experiment two WISTAR SPF(Specific Pathogen Free)/VAF(Virus Pathogen Free) male rats (age at the beginning 50 weeks) were exposed to a field specifically designed to modify calcium dependent enzymatic activities as indicated in the previous paragraph, for 35 days continuously. The animals were then observed without any other intervention for 182 days after the exposure. The limited number of animals used was due to the necessity of a constant exposure and the availability of a small set up. Furthermore, two animals of the same strain, used as controls, were kept in the same conditions without exposure.

In the main protocol, 13 male OFA (Oncins France Strain) SPF/VAF rats, from Charles River Laboratories Italia (Calco-LC), were exposed for a total time of 50 days.

Eight animals were 8–12 weeks old, and Eight animals were 68–72 weeks old. They were hosted into the cages according to the scheme: 4 adult rats in cage 1; 4 adult rats in cage 2; 2 aging rat in cage 3; 3 aging rats in cage 4; 3 aging rats in cage 5. After 15 days of adaptation, all rats were subjected to 1^st ^blood drawing (1^st ^day of the trial – field *OFF*). After this first drawing 2 adult rats and 1 aging rat died. From 2^nd ^to 29^th ^day, all rats were subjected to continuous irradiation by low intensity and frequency electromagnetic fields (ELF) (field *ON*); on 15^th ^and 29^th ^day respectively, 2^nd ^and 3^rd ^blood drawing was carried out. From 30^th ^to 36^th ^day ELF were deactivated (field *OFF*); on 36^th ^day 4^th ^blood drawing was carried out. From 37^th ^to 50^th ^day of trial ELF were reactivated (field *ON*); on 50^th ^day 5^th ^blood drawing was carried out. After the third and fourth drawing two other aging rat were lost. On 50^th ^day of trial all rats were sacrificed by intracardiac inoculation of embutramide + mebezonium iodide + tetracaine hydrochloride.

After each blood drawing the rats returned to the same cage. During the experiment, it was decided to submit to euthanasia subject 3 of cage 5 before the others for its health status, while rat 2 of cage 4 died twenty days before the end of the trial.

The individual body weight was controlled on 1^st^, 15^th^, 29^th^, 36^th^, and 50^th ^day together with blood drawing, and the amount of food consumed by each group was noted during the trial.

The data shown in the Figures are the averages on the animals which survived along the whole duration of the experiment, i.e. 6 adult and 5 aging rats.

### Post-mortem examination

A complete post-mortem examination of all the animals was performed after euthanasia and lungs, heart, spleen, liver, pancreas, kidneys, skeletal muscle and brain samples were fixed in buffered 10% formalin. Half of the brain, ocular tract and eyes were frozen and stored at -80°. Tissues were then processed in routine manner for histological evaluation, cut at 5 *μ*m thickness, and stained with H&E. Liver sections were also stained with periodic acid-Schiff (PAS)/diastase techniques to assess hepatic glycogen.

### Blood sample analysis

All blood samples were obtained by intracardiac drawing after anaesthesia with tyletamine hydrochloride and zolazepan hydrochloride combined with xilazine hydrochloride. Blood samples added to K_2_-EDTA were utilized to determine several haematology and biochemical parameters (see below). Blood sampling was performed in animals with no restriction to food availability. In order to reduce variability due to recent food uptake, care was taken to obtain samples at the same time of the day, namely between 3 and 4 pm, i.e. 7–8 hours after light start.

### Determinations of haematological and biochemical parameters

Haematological parameters (WBC, RBC, haemoglobin, hematocrit, MCV, MCH, MCHC, platelets, and differential leukocyte count: neutrophils, lymphocytes, monocytes, eosinophils, basophils) were measured in blood samples, added up with K_2_-EDTA, by ADVIA 120 HEMATOLOGY SYSTEM (BAYER Corp. Diagnostic Division, Tarrytown, NY, USA) provided with specific veterinary software for rats.

Biochemical parameters (Alanine aminotransferase (ALT), Aspartate aminotransferase (AST), Creatine kinase (CK), creatinine, cholesterol, glucose, total protein, tryglicerides, urea) were measured in plasma samples, obtained after centrifugation of blood samples added with K_2_-EDTA, by automatic analyser ROCHE HITACHI 912 PLUS (ROCHE Diagnostic Corp., Indianapolis, USA).

Fatty acids (palmitic acid (C16:0), stearic acid (C18:0), oleic acid (C18:1*ω*9), linoleic acid (C18:2*ω*6), linolenic acid (C18:3*ω*3), eicosatrienoic acid (C20:3*ω*9), arachidonic acid (C20: 4*ω*6), docosaesanoic acid (C22: 6*ω*3), and nervonic acid (C24: 1*ω*9)), derived as methyl esters, were measured in plasma samples according to Carnielli [16].

## Results

The first experiment was performed by exposing two animals to a field purposely designed to modify calcium dependent enzymatic activities as described in the previous part of this paper. The limited number of animals exposed was due to the necessity of a constant type of exposure and the availability of a small set up. Two animals of the same strain, used as controls, were kept in the same conditions without exposure. The exposed and unexposed animals were monitored and rapidly showed a different pattern of body weight and size. With a limited exposure (35 days) and a relatively long follow up (182 days), both animals reached a weight of over 900 grams, while the two control animals reached a value of about 400 grams.

After this exploratory experiment another experiment was performed by using animals of two different ages. In this case, exposure was not characterized by a unique frequency of exposure field, because of the disposal of the cages along the rack as discussed above, but a quite broad number of ions, including Ca^2+^, Mn^3+ ^and Co^3+ ^might be involved. Such a circumstance makes it more difficult to relate the effect observed on metabolism to a specific ion and, trough the ion to a specific metabolic reaction. Furthermore, exposure was applied for selected periods (ON) followed by a short periods without any exposure (OFF). Everything but the AC field was kept constant during ON and OFF periods. A control group could not be used in this experiment, thus variations between on and off periods were considered as a built-in control. The OFF periods are quite well visible as changes in the rate of the monitored issues: they signal a different adaptation time of the organism in case of an external perturbation. Furthermore, the strain of rats we used in this experiment are very well characterized in their physiological parameters, hence what we observed and reported here can be compared with a large amount of historical data. Among a number of parameters recorded, in this paper are only discussed which who showed striking variations.

**Figure 3:**
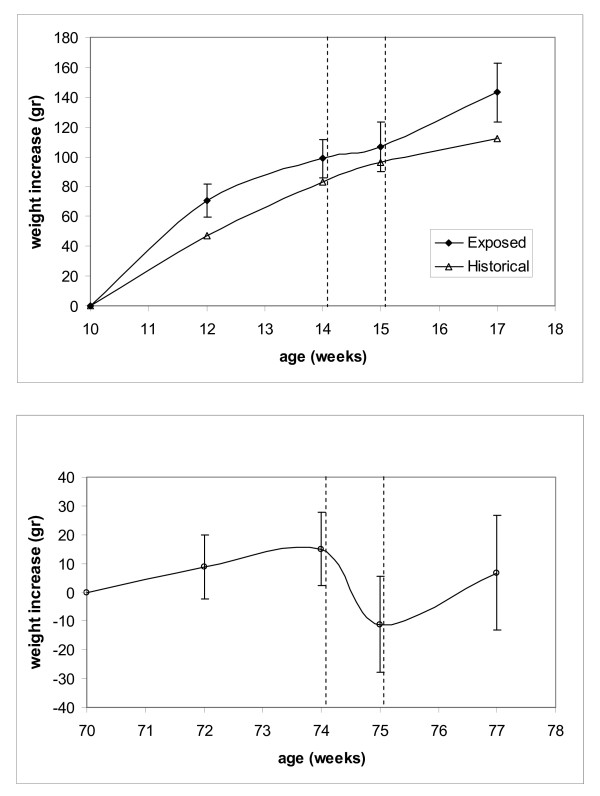
**Weight increase of OFA (Sprague Dawley) rats during the exposure to ELF weak magnetic field.** The abscissa shows the age of the animals in weeks. Panel (A): adult rats, the measured data are compared to historical data as available in the IFFA CREDO database for this strain; panel (B): aging rats, no weight increase is foreseen for this group. The region between the dashed lines shows the period of 1 week when the field was turned off.

With regard to body weight, the initial values observed were in agreement with the historical set (338 ± 19 gr. for adult and 599 ± 46 gr. for aging rats). Exposure appears to modify severely such an integrated parameter, as shown in Figure 3 thus confirming the results obtained in the first experiment. After 7 weeks of exposure (with 1 week of stop) the exposed rats show a weight increase much higher than the expected according to the historical data available in the IFFA CREDO database.

**Figure 4:**
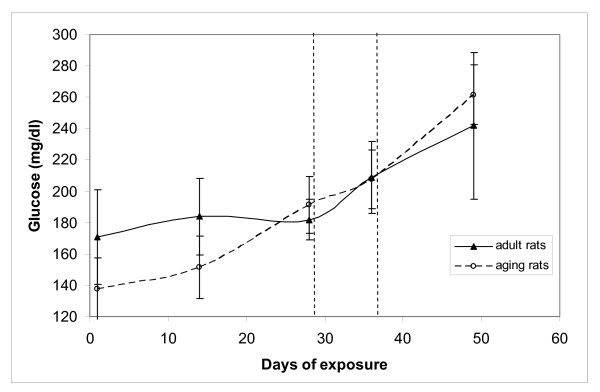
**Glucose content in blood of OFA (Sprague Dawley) rats during the exposure to ELF weak magnetic field.** Glucose levels were estimated at day 1,15, 29, 36 and 50. The region between the dashed lines shows the period of 1 week when the field was turned off. Error bars are ± 1 standard deviation.

Exposure appears to modify also blood glucose content, both in adults and in aging rats, as shown in Figure 4, where increased levels are evident at the end of exposure period. Since food was always available, no comparison with historical data obtained in fasting conditions can be provided. However fasting blood glucose do not change nor in adult nor in aging animals during a period of 7 weeks according to IFFA CREDO data base.

**Figure 5:**
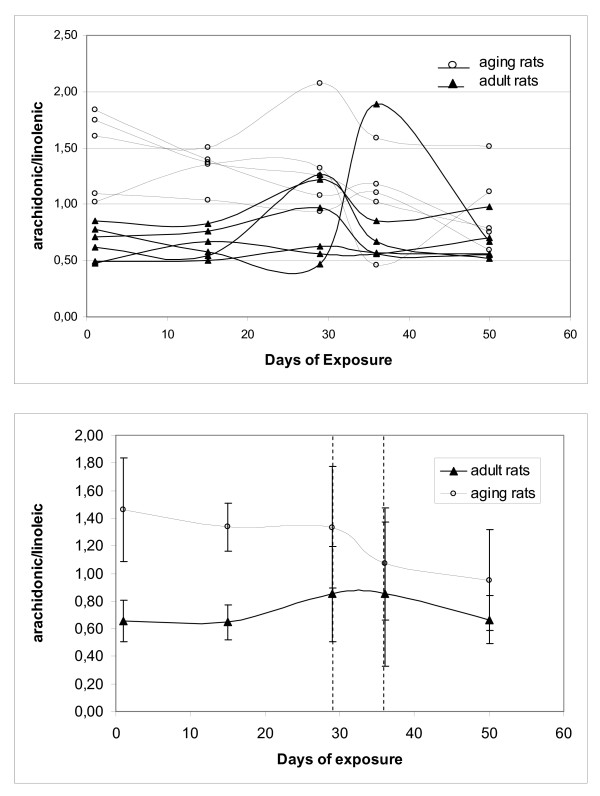
**Ratio between arachidonic and linoleic acid of OFA (Sprague Dawley) rats during the exposure to ELF magnetic field in adult and aging animals.** Data were estimated at day 1,15, 29, 36 and 50. The region between the dashed lines shows the period of 1 week when the field was turned off. Panel (A) shows the variation in each single rat: it can be observed the extreme variability of the individual response to the external stimulus enhanced for the aging rats. Panel (B) shows the averages of the panel (A) grouped in two subsets. Error bars are ± 1 standard deviation.

The effect of e.m. field on lipid metabolism is the most interesting finding of this research. Cholesterol and triglycerides appear to be only marginally affected by the stimulus applied, in fact, both parameters did not change throughout the experiment in both groups, however total free fatty acids and single fatty acids analysis showed visible changes. While exposure did not change substantially these parameters in adult animals, clear differences between the initial values and the final ones appear in aging animals. Figure 5 shows the ratio between arachidonic and linoleic acid during the experiment in adult and aging animals. In panel (A) can be seen the extreme variability of the individual response to the external stimulus, it is also interesting to observe that the major increase in the derivative of the values happens during the removal-addition of the magnetic field. Average ratios (panel B) were quite different at baseline, with much higher values for aging animals. They were substantially unchanged by exposure in adult rats, but reduced toward the values typical of the adults in aging rats during exposure periods.

With reference to haematology data, samples were available only for some of the rats, however in general terms we observed that leukocyte count tends to decrease during the experiment.

Finally, it is interesting to mention that at post-mortem examination no relevant gross findings were observed. In fact, most noticed lesions, in both age groups, are minimum blood collections in the pericardium (7 animals:) and in the thorax (5 rats). In 3 rats hemorrhagic findings were seen also in other sites. These findings are related to blood sampling and euthanasia.

Main findings of histopathological studies can be summarised as follows. A frequent finding was hepatic glycogenosis: PAS/diastase showed a mild multifocal staining in young animals (12 weeks old), while glycogen amount in hepatocytes cytoplasm was higher and diffusely distributed in 72 weeks old rats. Other changes did not show any significant difference between the two groups.

## Discussion

The ultimate goal of our research is the possible application of electromagnetic fields in the treatment of human and animal diseases, where ionic regulation of enzymatic activities have been altered and their restoration to normal is potentially useful for the patient. The present results give several helpful suggestions, both in terms of selection of possible patients and in terms of safety in order to reach this purpose. The present experimental work was carried out in order to have a coarse evaluation of the effects of the exposure, thus no defined experimental end point was selected a priori. Such an approach was mainly due to the fact that this kind of exposure had not been previously studied by other researchers and there is no available literature. This aspect not only makes it difficult to compare our findings with other results, but it also influenced the experimental design, preventing the possibility to choose in advance the most significant parameters to be measured. Nevertheless, the novelty of the observations and their relevance (especially the data about the weight increase) deserve, in our view, the preparation of the present report.

First, a huge effect in the growth of rats exposed to e.m. field having an amplitude of 40 *μ*T and a frequency of about 31 Hz is reported here for the first time. The animals exposed had their weight doubled as compared to the weight of control animals of the same strain, while no other negative effect has been observed in their physiology after 182 days of follow up and during the post mortem examination. This effect has been observed in two different experiments even though the second group was exposed for 50 days with an interval of 15 days (field OFF) and the weigh increase (about 150 grams) was observed only in adults and not in aged animals. The weight increase observed is clearly higher than expected on the basis of the historical data available for these particular strains in the IFFA CREDO database.

Second, changes in several metabolic parameters as a consequence of exposure to e.m. fields have been also observed. The frequency of the field was such to "stimulate" several ions in the sense of the cyclotron frequency. These ions may be involved in the enzymatic chains experimentally affected. This issue needs to be analyzed in detail in the future through well designed experiments.

Even though the data available are not exhaustive, because of the limited number of animals observed up to now, no major pathological signs related to exposure have been observed in post mortem analysis. Indeed, as far as authoptic findings are concerned, most of the findings described were noticed in both groups, independently from age, and they could be iatrogenic or aging-related. In fact, gross hemorrhagic lesions and some of the described microscopic changes (chronic pericarditis and myositis, pulmonary edema and CNS findings) are possibly related to intra-cardiac blood sampling and to injections of anaesthetic drugs. In particular, changes observed in nervous tissues could be due to an early mild hypoxic condition [17].

From a regulatory point of view, the intensity of the fields applied is considerably below the threshold risk value, our preliminary new data seem to agree with this view and at present there is no reason for concern. From the point of view of patient selection, our findings cannot be directly extrapolated to human pathologies. In general terms, aged animals appear to be more sensitive to the exposure: major changes have been observed in this group and not in the adult animals. In particular the derivatives of the individual responses are much higher in the ON-OFF transient especially for aging animals, In order to explain these observations, we suggest that adult young animals have the capability to reach homeostasis even in the presence of external stimulation, like the one we applied, while aging animals have a reduced capability to cope with external stimulation and thus measurements of several variables are likely to reveal alterations.

By analyzing the observed changes in aging animals, it can be said that: a) body weight increased during the first and the second period of exposure and decreased in the off period this results confirms the first experimental run described; b) blood glucose substantially increased in this group more than in young animals; c) the circulating levels of specific free fatty acids clearly show the effect of the exposure.

It is interesting to mention that in a recent paper Torres-Duran et al [18] showed an action of exposure on lipids also with a short exposure, namely 24 hours, pointing out the importance of lipid metabolism as one of the main targets to analyse in order to understand the overall effect of exposure.

Knowing that the total free fatty acid content of the blood may be affected substantially by stress, the data were analyzed as the ratio between different fatty acids, in particular as the ratio between single acids and their metabolic precursors. By applying this kind of analysis, the most interesting findings were related to arachidonate/linoleic ratio, most likely a direct consequence of the enzymatic activity of elongase and desaturase. The baseline of aging rats showed substantially higher values than in adults, likely indicating enhanced conversion, furthermore, exposure did not substantially modify the values in adult rats, while decreased the ratio in aging rats, thus bringing the ratio closer to that observed in adults. The importance of such a difference in the comparison between adults and aging rats is further outlined by the fact that other calculated ratios, such as eicosatrienoic/oleic acids ratio and docosaenoic/linolenic acids ratio, were different neither at baseline, nor after exposure.

## Conclusion

In conclusion, we provide initial evidence that long term exposure to well defined electromagnetic fields may have relevant effects in mammals, thus affecting parameters like body weight, blood glucose and fatty acid metabolism. The present findings have to be confirmed and extended to fully understand their importance; nevertheless, they indicate that this line of research may lead to important results. The clinical consequences of these observations cannot be precisely estimated yet, even tough they point out towards a rational target for possible therapeutic interventions, to be analysed in further studies.

## Competing interests

Authors disclose any financial and non-financial competing interests (political, personal, religious, ideological, academic, intellectual, commercial or any other) related to the publication of the manuscript

## Authors' contributions

GG carried out the experiments and helped to draft the manuscript. ADN analyzed the results and drafted the manuscript. MP analyzed the results and drafted the manuscript. VF carried out the experiment and helped to draft the manuscript. FB supervised the electronic equipment. SM carried out the histopathological studies. DB analyzed the results and helped to draft the manuscript. GT conceived of the study, and participated in its design and coordination. All authors read and approved the final manuscript
